# Glossitis

**Published:** 1849-03

**Authors:** 


*Glossitis.* By GEORGE GRIFFIN, Esq. Surgeon, Half-Pay 85th Regiment, Quebec.—Within the last two or three days, I have seen in the Medical Journal under your superintendence, the very interesting case of Miss M. as furnished by Dr. Marsden. I am of opinion that it is, in some sort, the duty of every medical man, to lend what aid he can in the investigation of rare and dangerous diseases, more especially, and to specify what experience may have taught him. Therefore, in furtherance of that conviction, I am induced to consider that perhaps this communication may not be uninteresting to your readers.

It has been my fortune to see but two of these cases during a service of upwards of thirty-five years, and they are well impressed upon my mind. The first was in the person of a Neapolitan sailor, while I was on detach-

---

* The italics are my own, and intended to impress these points upon the reader.

ment in one of the Ionian Islands, in the summer of 1822. The man was landed from a gun-boat, without any history of his state, further than that he complained, for the first time, about twenty-four hours previously to the vessel making the island.

I found the tongue enormously enlarged, protruding from between the teeth; breathing hurried, anxious, difficult, performed altogether through the nostrils; suffocation impending; face livid; eyes protruding; moaned incessently, and very restless. The accompanying fever purely inflammatory; heat of the skin intense; a powerful pulse, and strong action of the carotid and temporal vessels; he was sensible; could not articulate, but expressed his distress by signs. Blood was taken from the arm without delay, in no stinted degree; the bowels freely opened by active medicines; but there was scarcely time to carry these remedial measures into effect, before it became abundantly evident, that, unless relieved, the man must die, and that shortly. I had no one to advise with, and had never seen or heard of the disease before, however, there was no alternative, and the mode of relief evident; having secured the head from moving, I depressed the lower jaw and tongue, with a strong iron spoon, as much as practicable, and then introduced a scalpel sideways or flat, as far back as I could, and made two incisions into the dorsum of the tongue, from behind forwards to the apex, one on each side.

The hœmorrhage was very profuse, but the relief immediate; no purulent matter followed the incision. So rapid was the declension of the swelling, that the next day the place of the incisions could scarcely be detected; those who have witnessed lacerated wounds of the tongue, must have remarked this circumstance. A week or two afterwards, I had a second case under my care, also a foreign sailor. Now aware of the nature of the disease and its danger, I no longer hesitated, but made the incision at once; both these men did well. The after treatment was small doses of some mercurial preparation to affect the mouth. I am writing of occurrences twenty-six years ago—I do not know that I should have resource to mercury now; the result, however, was quick recovery.

The father of the young lady whose case is related by Dr. M——, has been well known to me for several years—his daughter from childhood. Some time in August last, we met accidentally, and he narrated to me his daughter's perilous state, and the circumstances attending it. I went to see her the following day, and she expressed to me the immediate relief she had experienced from "the incision," and her distressed state previously. She was, as might have been expected, in a state of great debility, but recovering. The tongue was, though large, generally soft, and of a dull leaden color. As well remarked by Dr. Marsden, "The disease is so rare, that it usually takes the surgeon quite by surprise."—*Ibid.*

ESPLANADE, QUEBEC, NOV. 20, 1848.

